# Case reports – common and external carotid artery resection in head and neck cancer patients

**Published:** 2013-06-25

**Authors:** B Popescu, SVG Berteșteanu, R Grigore, R Scăunașu, S Voiculescu, CR Popescu

**Affiliations:** * E.N.T. Department, Coltea Clinical Hospital, Bucharest, Romania; ** General Surgery Department, Colțea Clinical Hospital, Bucharest, Romania

**Keywords:** malignancy, chronic brain ischemia, oncological margin, neck dissection

## Abstract

Most head and neck cancer patients are first referred to an E.N.T. specialist thus the need for that surgeon to be the leader of the multidisciplinary team. Oncological surgical interventions need to ensure clear resection margins; this means that whatever anatomic structures are involved in the tumor spread, need to be resected. The carotid artery is a vascular vessel system that provides blood supply for the head and neck region, the most important structure being the brain and its organs. The ligation or the resection of the common carotid artery leads to an abrupt decrease of blood flow towards the brain, which can cause single sided paralysis, decreased cognitive functions, shock and even death. Common or internal carotid arteries ligatures or resections can be performed in patients with malignant tumors of the head and neck. This is a synopsis of 2 successful cases of patients who underwent common and external carotid artery resection.

## Introduction

Head and neck cancer is one of the most important public health concerns worldwide because of the ever increasing number of tobacco and alcohol consumption, two of the major risk factors for this pathology. This is the perfect example for a multidisciplinary patient approach in modern medicine in which case specialists of different areas of expertise need to collaborate. Most head and neck cancer patients are first referred to an E.N.T. specialist thus the need for that surgeon to be the leader of the multidisciplinary team. The complex therapy of the head and neck cancer involves the presence of a vascular surgeon or an E.N.T. specialist who is capable of dealing with vascular disease entities. The vascular system of the head and neck is of outmost importance due to the implications on the local anatomy and on the cerebral functions. The lateral region of the neck, starting from the clavicle and ending at the base of the skull is the site of a possible malignant tumor, which may involve the carotid system.

 The tumor spread explained by the lymphatic and vascular spread in most cases finds its local development towards the large vessels of the head and neck. This metastasis process is to alter whatever anatomical elements of the neck may be in the pathway of the tumor. Lymph node metastasis is considered a loco-regional metastasis of the tumor process. They are in direct relation to the large vessels of the neck, the internal jugular vein the carotid system, and also to the nerves of the neck, most important to the vagus nerve, hypoglossal nerve and the cervical sympathetic trunk. Oncological surgical interventions need to ensure clear resection margins; this means that whatever anatomic structures are involved in the tumor spread need to be resected. Neck dissection is a topic of clear indications and classifications that were established by The American Academy of Otolaryngology-Head and Neck Surgery (AAO-HNS) published in 1991 and revised in 2001 [**1**]. The other organs, vessels, nerves of the head and neck that are involved in the tumor process also need to be resected in order to ensure the surgical oncological effort.

### Background

 The carotid artery is a vascular vessel system that provides blood supply for the head and neck region, the most important structure being the brain and its organs. The ligation or the resection of the common carotid artery leads to an abrupt decrease of blood flow towards the brain, which can cause single sided paralysis, decreased cognitive functions, shock and even death. This procedure can be performed because of the presence of the collateral arterial vessels that can supply blood to the brain and organs through the arterial circle of Willis [**2**]. This arterial anastomosis is the basis for performing such carotid resection or ligation. When the tumor process is slowly developing and affecting one or more carotid arteries (common, internal or external carotid arteries) there is an increased possibility of developing higher blood flow through the contralateral carotid system and through the vertebral arteries. This means that the tumor process acts like a trigger for the development of the collateral blood flow supply. 

 When ligating the common carotid artery on one side of the neck there is the possibility that the patient will recover the entire brain and organ functions due to a retrograde blood flow through the external carotid artery and into the internal carotid artery, this being possible due to the Willis arterial circle. Still, there is a possibility that the patient will develop chronic brain ischemia or even die which makes this a high-risk procedure. 

 In the 19th century, JA Wyeth performed some 800 common artery ligations, 300 of which resulted in the death of the patient. He realized that the supraomohyoid region of the common carotid artery is the best site to perform the surgical procedure. The structure of the carotid systems includes the existence of baroreceptors, chemoreceptors and nerve plexus in the bifurcation region of the common carotid artery [**3**]. These essential anatomy data are the reason for light manipulation of the carotid system during surgical procedures at this site; otherwise, there is the possibility of reflex bradycardia, drop in the arterial blood pressure, syncope, cardiac arrest or even death.

 Several studies made on young rat models have indicated that regions in the brain suffer from acute blood flow hypoperfusion following the ligation or resection of the carotid system (common or internal carotid artery), but it is likely to have a quick self-recovery, within 3 weeks time. The results of the studies suggest that the blood vessels in young rat models have plasticity to external insult [**4**]. When analyzing our patients we found that all 5 had a full recovery, motor and cognitive functions, within a month after the surgery. 

 There are other studies on rats that revealed the significant impairment in the eight-arm radial maze task in rats, following internal carotid artery ligation, only than in common carotid artery-ligated rats. The impaired learning process of the young rats led to the conclusion that the reduction of the blood flow might be an important risk factor for the appearance or the exacerbation of the cognitive decline in dementias [**5**].

The indications for common and external carotid arteries ligation or resection include [6,7]:

 - neck trauma caused by a sharp or blunt foreign body, firearm projectiles, 

 - arterial aneurysm, 

 - carotid dissection,

- Takayasu’s arteritis, which is uncommon at this site, but may still develop aneurysms of the carotid system,

- radiation arteritis with the rupture of the carotid arteries,

- salivary fistulas which can lead to the rupture of the carotid system

- massive hemorrhage in the irrigation territory of the external carotid artery,

- malignant process of the neck that includes the carotid system,

- tumor of the carotid corpuscle,

- artery thrombosis secondary to near-site infections

- preliminary stage in high hemorrhage surgical interventions of the head and neck region such as tongue tumor, maxillary cancer, fibroma, (in these situations there is the need for external carotid artery ligation).

- essential epilepsy when there might be the need for denervating the reflex region of the carotid sinus.

There are no absolute contraindications when dealing with a life-threatening situation [**8**]. Still, there are situations when the ligation or the resection of the carotid system should better not be performed such as inoperable cases, life-threatening comorbidities, atherosclerosis which increases the risk of an ischemic attack. 

The patients’ medical condition is to be assessed by the multidisciplinary medical team and a firm surgical indication must be made. 

### Case reports

 Case I

 A 58-year-old, male, from the urban environment, heavy smoker – 1 pack of cigarettes per day for 25 years, alcohol consumer, referred to Coltea Clinical Hospital, E.N.T. Department for dysphagia, odynophagia, left ear pain and the presence of a tumor mass of the neck, which he stated that appeared about 6 months prior to the examination, with a slow and progressive evolution. The patient is known to have high blood pressure, maximum value of 210 mmHg, under treatment, and without any other significant pathology.

 When performing the clinical exam we found an ulcerated tumor mass in the hypopharynx, referring to the left lateral wall of the pharynx and to the root of the tongue. The tumor process extended to the lateral region of the neck involving the submandibular gland, the levels II-V lymph nodes, large vessels of the neck and skin. Imaging studies confirmed the extension of the tumor process. The Doppler ultrasound examination showed a near to 0 blood flow through the external carotid artery and its branches. After performing a biopsy from the tumor under local anesthesia – invasive squamous cell carcinoma, we informed the patient about the choices of therapy and obtained his informed consent to perform the surgical intervention.

**Fig. 1:**
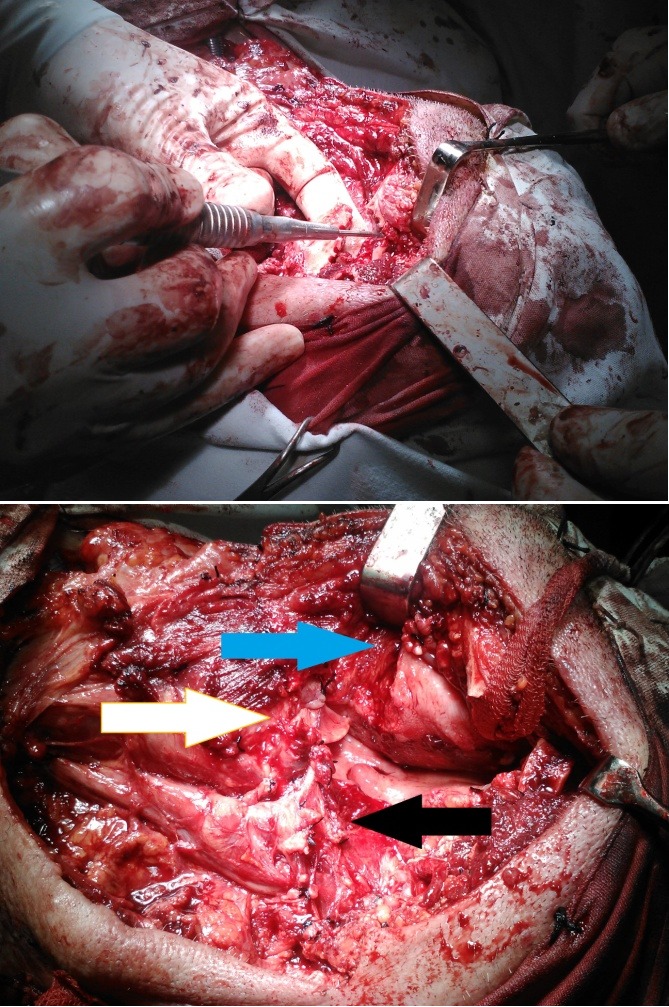
Left-lateral mandibulotomy. Right blue arrow – root of the tongue, white arrow – epiglottis, black arrow – resected external carotid artery and its branches

 We performed a tracheostomy in order to ensure a maximum access to the oro and hypopharynx, radical neck dissection with the resection of the internal jugular vein, accessory spinal nerve, sternocleidomastoid muscle, entire fatty tissue from the clavicle to the base of the skull. In order to get the access to the entire tumor mass we needed to perform a lateral mandibulotomy (**Fig. 1**). The resection piece included the root of the tongue, lateral wall of the pharynx, the submandibular gland, lymph nodes levels II to Va and skin. 

**Fig. 2:**
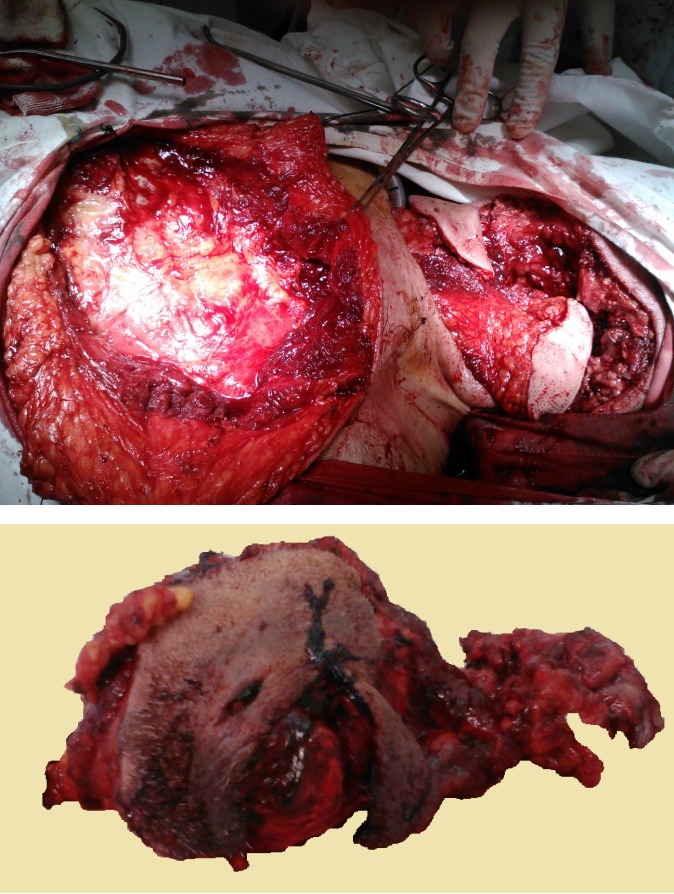
Left-pectoral musculocutaneous pedicle flap rotated to the lateral region of the neck. Right–resection piece with affected overlaying skin

 The pharynx was primarily closed with silk sutures and the continuity of the mandible was made by plates and screws osteosynthesis. The remaining defect was then closed with a pectoral musculocutaneous pedicle flap rotated to the lateral region of the neck (**Fig. 2**). Side-by-side simple sutures then closed the pectoral region. Drain tubes were placed in the pectoral region and the lateral neck region and also a naso-gastric feeding tube. The drain tubes were removed after 7 days. Enteral feeding was started the first day after surgery and continued for 19 days. After performing a Gastrografin exam of the digestive system that concluded there were no fistulas, we removed the nasogastric tube and the patient started to feed orally. 

 Follow-up

**Fig. 3:**
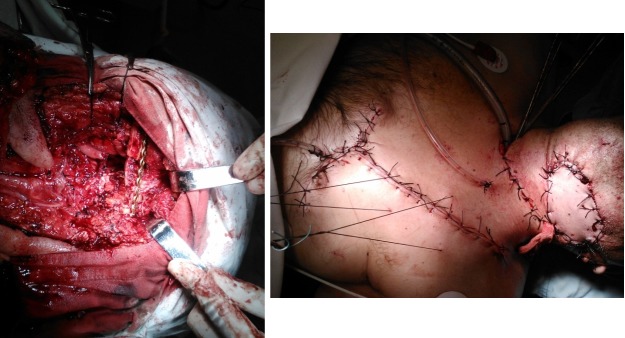
Left – Osteosynthesis with plates and screws. Right – postoperative aspect

 The evolution of the patient was very good with a 6 weeks postoperative wound normal aspect and relapse free. There is no need for the plate and screws to be removed in a second stage surgery (**Fig. 3**). The patient started external beam radiation therapy at 8 weeks after the surgery and finished it without significant side effects. The 6 months stage control revealed no sign of relapse and good wound healing. 

 Case II

 A 71-year-old male, from urban environment, smoker – 1/2 pack of cigarettes per day for 32 years, occasional alcohol consumption, referred to Coltea Clinical Hospital, E.N.T. Department for the appearance of a tumor mass in the lateral region of the neck, that appeared 1 year prior to the specialist exam, dysphagia, weight loss of 10 kg in the past 5 months. The patient had diabetes mellitus in treatment with oral antidiabetic drugs. 

 Blood tests revealed an inflammatory syndrome with an erythrocyte sedimentation rate of 24 mm/h and a fibrinogen count of 575 mg/dl and a slightly altered coagulation profile. Imaging studies revealed the presence of the tumor mass in the lateral region of the neck and an ulcerated tumor mass in the thyro-hyoepiglottic lodge. The biopsy from the larynx was positive for infiltrative poorly differentiated squamous cell carcinoma. Panendoscopic examination led to the surgical indication of partial pharyngectomy total laryngectomy, right radical neck dissection and left functional neck dissection.

**Fig. 4:**
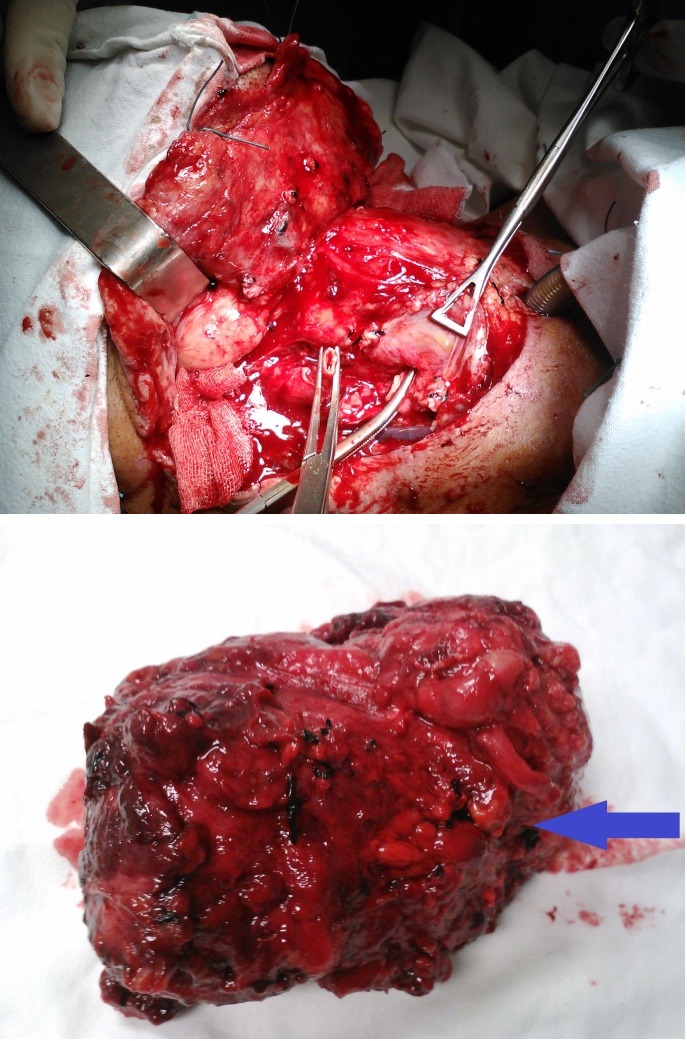
Left radical neck dissection, resection of the common carotid artery, right – resection piece from the left lateral region of the neck, blue arrow shows the common carotid artery resected

**Fig. 5:**
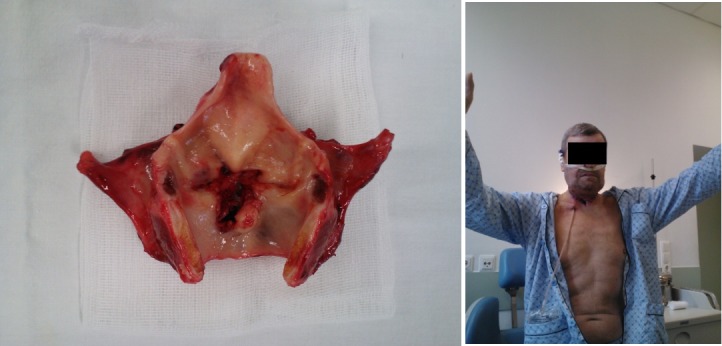
Left – larynx, right – 6 days after surgery

 We operated on the patient removing the tumor by performing a total laryngectomy with partial pharyngectomy, right submaxilectomy, right radical neck dissection, resection of the common carotid artery, resection of the external carotid artery and a tracheostomy (**Fig. 4,5**). 

 We placed a drain tube in the right lateral region of the neck and a nasogastric feeding tube.

 Follow-up

 The patient was admitted in the ICU for 2 days in order to monitor his medical condition. There were no early complications. Motor functions, cognitive functions or judgment were not impaired after the surgery. The drain tube was removed after 5 days and the sutures after 11 days. Enteral feeding was started 12 hours after the surgery with the removal of the nasogastric tube at 17 days after the surgery when oral feeding was restored. The patient underwent external beam radiation starting at 10 weeks after the surgical procedure with the appearance of dryness in the mouth and the reddening of the skin. The patient was clinically examined every other 2 months with a good evolution of the wound and without any other impairment for the last 8 months. 

## Conclusions

Head and neck malignant tumors have an increasing incidence probably because of the increased tobacco and alcohol consumption and the association between the two. Patients who refer to the specialist have a poor social and economical status. In terms of medical care, this is reflected in the late stages of the disease when presenting to the specialist. The local metastasis of the malignant tumor disease may involve all regions of the neck, subsequently the carotid system. The histological structure of the artery, mainly the fibrous layer, should protect the vessel from the malignant disease. Still, in late stages, the alteration of the anatomic structures leads to the extension of the vessel towards the lumen. 

The ligation of the common carotid artery is a surgical procedure that has many complications, but should be performed in patients for whom the oncological committee approved the surgical procedure. Good diagnostic leads to good decisions and thus increasing patients’ chances of survival. Although we are discussing about a poor prognosis in stage IV cancer patients, the surgical treatment should not be denied to any patient who is willing to undergo such radical surgical oncological intervention. We also need to have an extensive discussion with the patient and relatives, if the patient desires, about the accidents, complications, follow-up and prognosis in order to allow the patient to have a complete view on the disease. 

 The medical consent form and the informed consent of the patient are necessary as well as the approval of The Ethics Committee of the Hospital. These patients have a poor quality of life and need special attention for individual and social rehabilitation after the surgery [**9**]. We need to ensure the quality of life for these patients as this is known to improve the healing process and the social rehabilitation takes less time.

 Common or internal carotid arteries ligatures or resections can be performed in patients with malignant tumors of the head and neck considering that the tumor process is a slow one and thus slowly reducing the blood flow through these vessels. If the imaging studies reveal a blood flow through the vessels and there is a need of resecting the arteries than a specific mechanical device (the Ghitescu forceps) should be used to slowly and progressively decrease the lumen of the vessel. After the sufficient decrease of the blood flow, which allows the contralateral arterial circulation to gain pressure, the resection of the common or internal carotid arteries can be performed.

 The earlier onset or the exacerbation of dementia in patients with carotid system ligatures or resections is still a subject of debate, but this needs to be taken into consideration in the follow-up of the patients and the families need to be warned about this possible late complication.
